# The Evolution of Same-sex Sexual Behavior: Using Old Theory to Answer New Questions

**DOI:** 10.1093/icb/icag015

**Published:** 2026-03-25

**Authors:** Brian A Lerch

**Affiliations:** Center for Population Biology, University of California, Davis, CA 95616USA

## Abstract

The evolution of mating preferences and the consequences of the resulting sexual selection are intensively studied topics in evolutionary biology. Nevertheless, until relatively recently, the evolution of perhaps the most fundamental of all mating preferences, the preference for mating with opposite-sex individuals, has received little attention. In the past two decades, however, there has been an explosion of literature on the evolution and expression of same-sex sexual behavior (SSB) in nonhuman animals. Here, I show that literature on SSB has strong connections to many insights from classic theory on mate choice. I argue that explicitly integrating this “old theory” on mate choice proves tremendously informative for understanding the evolution of SSB. To do so, I review what is known about the expression and evolution of SSB. I begin by explaining why selection may favor imperfect sex discrimination (i.e., the failure to accurately assess the sex of potential mating partners), how imperfect sex discrimination manifests in SSB empirically, and the origin of sex discrimination. I then review experimental studies on how varying social conditions, such as density and sex ratio, influence the plastic expression of SSB. Next, I turn to evidence for adaptive SSB, wherein the act of engaging in sexual behavior with same-sex conspecifics carries fitness benefits. I conclude by arguing that connecting results on SSB to “classic” work on mating preferences leads to a deeper understanding of how SSB evolves.

## Introduction

*“If I have seen further it is by standing on the shoulders of Giants.”*

- *Isaac Newton*

Why do animals prefer to mate with some conspecifics over others? This question has puzzled biologists for well over a century. Many possible answers have emerged and are central to the evolutionary study of mate choice. In most systems with a mating preference for a specific trait, a correlation forms between the genes controlling the preference and those controlling the trait. Consequently, if preferences drive an increase in the frequency of the trait, they too will increase in frequency due to indirect selection (the “Fisher process”; Fisher 1930; Lande 1981; Kirkpatrick 1982; Prum 2010; Xu et al. 2023). Preferences need not be for arbitrary traits, however, and may be more likely to evolve for traits that indicate an organism’s quality, fit to their environment, or compatibility with a given mate (Zahavi 1975; Hamilton and Zuk 1982; Møller and Alatalo 1999; Puurtinen et al. 2005; Dhole et al. 2018; Yan et al. 2024). In addition to these indirect benefits of mate choice (Fry 2022; Servedio 2025), mates may provide direct benefits to choosers, such as enhanced parental care (Hill 1991; Price et al. 1993; Alonzo 2012). Finally, mating traits may be favored by exploiting preexisting sensory biases from other contexts, like foraging (Endler 1998; Rodd et al. 2002; Fuller et al. 2005; Liu et al. 2023). Clearly, there exists a strong conceptual foundation for understanding why animals prefer to mate with some conspecifics over others.

Under what conditions, and why, do animals prefer to mate with opposite-sex conspecifics over same-sex conspecifics? Until relatively recently, this question was rarely asked. That is, although the evolution of mate choice has been extensively studied for decades, the preference for mating with opposite-sex individuals was tacitly assumed and taken for granted. It is not without reason that biologists largely neglected to study preferences for mating with members of the opposite sex. Indeed, barring some exceptions discussed below, only matings with the opposite sex result in reproduction, providing a clear advantage to developing an opposite-sex mating preference. However, these opposite-sex mating preferences must themselves evolve (Parker 2014; Monk et al. 2019). Understanding the evolution of opposite-sex mating preferences (i.e., the evolution of sex discrimination; Box 1) and its implications therefore provides insights into a fundamental and widely shared feature of mate choice.

Box 1. **Glossary.**

*Adaptive same-sex sexual behavior*: Cases of SSB where expending mating effort on same-sex individuals per se improves an organism’s ability to survive or reproduce

*Imperfect sex discrimination*: Not courting and mating exclusively with members of the opposite sex due to a failure to accurately assess, or attempt to assess, the sex of one’s partner

*Indiscriminate mating*: Mating with no attempt to discern the sex of one’s partner

*Mistaken identity*: Cases of SSB occurring due to imperfect sex discrimination

*Same-sex sexual attraction*: Courting and mating preferentially with members of the same sex

*Same-sex sexual behavior (SSB)*: Any behavior between members of the same sex that could lead to fertilization when engaged in with members of the opposite sex

*Sex discrimination*: Courting and mating preferentially with members of the opposite sex

The evolution of sex discrimination is also interesting because strict and universal opposite-sex mating preferences are less prevalent across animals than has long been believed. Same-sex sexual behavior (SSB) is commonly observed in many forms and across a wide range of taxa, including birds (Poiani 2008; MacFarlane et al. 2010; Gillies and Siddiqi-Davies 2025), mammals (Vasey and Jiskoot 2010; Furuichi et al. 2014; Sugita 2016; Gómez et al. 2023), reptiles (Shine et al. 2001; Rodrigues and Liu 2016), mollusks (Ambrogio and Pechenik 2008; Hoving et al. 2019), echinoderms (Young et al. 1992; Slattery and Bosch 1993; McCarthy and Young 2002), insects (Van Gossum et al. 2005; Maklakov and Bonduriansky 2009; Scharf and Martin 2013; Hoskins et al. 2015; Rayner and Bailey 2019), and other invertebrates (Palma-Onetto et al. 2026). Historically, little attention was paid to understanding the evolution of SSB, likely related to cultural biases about what was viewed as acceptable and/or anomalous (Bailey and Zuk 2009; Monk et al. 2019). However, in the last two decades, a great deal of work has been devoted to understanding the prevalence and evolution of SSB across animals. Recent evidence that SSB has a heritable genetic basis (Han and Brooks 2015*a*; Clive et al. 2023) underscores that an evolutionary perspective is needed.

I define SSB to be any behavior between members of the same sex that could lead to fertilization when engaged in with members of the opposite sex (Box 1). This definition is deliberately agnostic to whether the individual initiating SSB perceives the function of the interaction as reproduction. Given its different manifestations, determining what, exactly, counts as SSB is far from straightforward. Indeed, the formation of a same-sex pair bond in birds seems qualitatively distinct from virgin male *Drosophila* courting other males. Defining the scope of SSB is, however, not my goal and I simply note that I spend more time discussing behaviors that are most directly relevant to reproduction (like courtship and copulation) compared to behaviors further removed from reproduction (like pair bonding) that I consider only briefly. My review considers obligately outcrossing, gonochoristic animals and does not provide insights into human sexuality, which is influenced by sociocultural factors that I will not discuss. I also will not focus on same-sex sexual attraction (Box 1), a topic I return to under “Future Directions,” largely because most work on same-sex sexual attraction has been carried out exclusively in humans and focuses on mechanistic, rather than evolutionary, drivers. Finally, I note that this review is not intended to be comprehensive but rather highlights specific work on the evolution of SSB that helps to clarify how SSB relates to classic theory on mate choice.

I begin a review of our understanding of the evolution of SSB by discussing the evolution of sex discrimination, both in contemporary taxa and at the origin of sexual behavior. I then review empirical evidence for the expression of SSB plastically responding to different social conditions, especially from manipulative work in insects. I next discuss potential adaptive benefits to SSB in both invertebrate and vertebrate taxa. Finally, I discuss future directions and outstanding challenges for the evolutionary study of SSB. Throughout, I draw parallels between the expression and evolution of SSB and the expression and evolution of mate choice from among opposite-sex individuals (summarized in Table 1). My primary thesis is that it is important to connect studies of SSB to classic work on mate choice and sexual selection. Such “old theory” can be tremendously informative when applied to SSB.

**Table 1:** Analogs between classic theory on mate choice from among opposite-sex individuals and SSB

Factor influencing mating	Result from classic theory	Result from SSB
Costs to choosiness	Costs promote random mating	Costs promote SSB
Matings costs	Costs reduce the mating rate (e.g., through resistance)	Costs disfavor SSB
Opportunity costs	Risk of going unmated promotes random mating	Risk of missing a mating promotes SSB
Encounter rate	Infrequent encounters promote random mating	Infrequent encounters promote SSB
Bonding	Mating facilitates pair-bond formation	SSB can enhance prosocial coordination
Rearing young	The need for biparental care leads to pair-bond formation	Same-sex pairs provide biparental care without paternal care
Acceptance threshold	Factors lowering the acceptance threshold lead to permissive mating decisions	Factors lowering the acceptance threshold lead to SSB

## The evolution and origin of sex discrimination

**Fig. 1:**
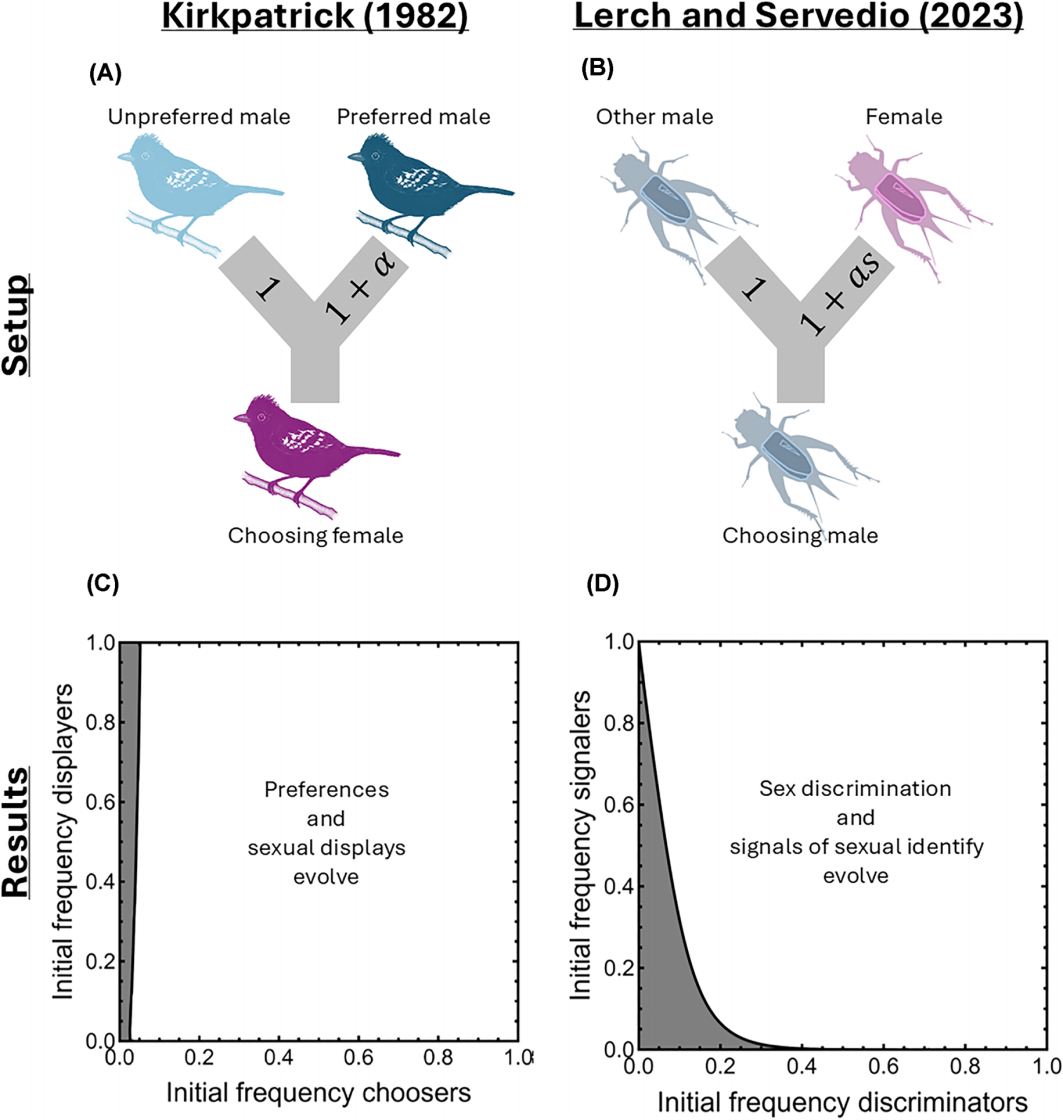
Comparison between a classic model of female choice for preferred versus nonpreferred males (Kirkpatrick 1982) and a model of sex discrimination (Lerch and Servedio 2023). (A and B) Both models assume that mate choice is frequency dependent and parameterize preference strength $\alpha $ or the strength of sex discrimination *a* assuming that these traits alter choice probability in a y-maze test. In Kirkpatrick (1982), a female (pink) is assumed to choose between preferred (dark blue) and nonpreferred (light blue) males. In Lerch and Servedio (2023) a member of the searching sex (blue, shown as a male in the Fig. for consistency with empirical studies) chooses between members of the same versus opposite (pink) sex. Here, an additional trait of the opposite sex *s* controls how readily the two sexes can be differentiated. (C and D) Comparison between results from the classic model with costs to choice and mutation on the display added (see Supplementary code) and the model of SSB. The frequency of individuals engaging in mate choice is on the horizontal axis. In the classic model, this is the frequency of females that express preferences versus mate randomly. In the SSB model, this is the frequency of searchers that display sex discrimination versus mate indiscriminately. The frequency of individuals signaling is on the vertical axis. In the classic model, this is the frequency of males that express a sexual display. In the SSB model, this is the frequency of targets that display a signal of sexual identity. White indicates that an equilibrium with both nonrandom mate choice and signaling/displaying evolves, whereas gray indicates that random/indiscriminate mating and no displaying/signaling evolve. In both models, a benefit to signaling/displaying depends on nonrandom mate choice, whereas a benefit to nonrandom mate choice only depends appreciably on the frequency of the signal in the SSB model

Many proximate and ultimate hypotheses have been proposed to explain SSB (Bailey and Zuk 2009). A large body of work on SSB focuses on imperfect sex discrimination as a proximate explanation. I begin the review by discussing the evolution of imperfect sex discrimination before moving to adaptive explanations for SSB. I define sex discrimination to be courting and mating preferentially with members of the opposite sex (Box 1). Viewed in this light, sex discrimination is analogous to preferences in classic sexual selection theory, with the strength of sex discrimination corresponding to preference strength. Indeed, formal population-genetic models of sex discrimination use the same form as classic population-genetic models of mating with preferred versus nonpreferred opposite-sex partners (Fig. 1; Kirkpatrick 1982; Lerch and Servedio 2023). In this section, I review the factors suggested to favor the evolution of imperfect sex discrimination and link them to factors promoting random mating in classic theory on mate choice.

If individuals do not or cannot express perfect sex discrimination, then SSB will occur. Broadly, SSB resulting from imperfect sex discrimination or, in the extreme case, indiscriminate mating (i.e., mating with no attempt to discern the sex of one’s partner) is said to occur due to “mistaken identity” (Harari et al. 2000) (Box 1). Before exploring what is known about the evolution and origin of sex discrimination, it is worth first unpacking the term “mistaken identity.” As noted elsewhere (Richardson and Zuk 2023; Richardson et al. 2024), the mistaken identity hypothesis does not imply that imperfect sex discrimination per se is maladaptive. Rather, the term applies to a proximate hypothesis that suggests SSB is not the deliberate choice or intention of the individual’s initiating the mating (Lerch and Servedio 2023). Thus, “mistaken identity” is, in my view, a misleading name as it could imply that individuals should be motivated to avoid SSB as an adaptation: an implication that, as we will see below, is often erroneous due to tradeoffs and constraints associated with mating decisions that result in the maintenance of SSB as a byproduct.

I now turn to considering the evolutionary causes of imperfect sex discrimination. Many costs, tradeoffs, and constraints have been proposed that could lead to selection for imperfect sex discrimination. For example, the strength of sex discrimination has been observed trading off with the total mating rate (Vasey et al. 2008; Logue et al. 2009; Han and Brooks 2015b). Here, SSB is maintained as a byproduct of a high sex drive. In another example, Parker (2014) developed a theory showing that a tradeoff between sperm production and an individual’s ability to move toward opposite-sex conspecifics can maintain imperfect sex discrimination. There exists some direct and indirect evidence of this tradeoff (Run et al. 1988; Matsumura et al. 2019).

Theory also showed that direct costs to sex discrimination (i.e., viability selection disfavoring individuals with stronger sex discrimination) can maintain imperfect sex discrimination (Lerch and Servedio 2021), a result that strongly connects to choosiness being less likely to evolve under direct costs to choice in classic models of mate choice (Pomiankowski 1987; Bulmer 1989) (Table 1). Classic theory suggests that choosiness can be maintained despite direct costs to choice if choosers are picking from among individuals that differ in their ability to produce viable offspring (Pomiankowski 1987). Such direct benefits arise naturally in the context of SSB because choosers are deciding between matings that can produce offspring (with the opposite sex) and those that cannot (with the same sex), suggesting that the evolution of sex discrimination should be more prone to overcoming direct costs than the evolution of choosiness between opposite-sex individuals. There is, however, no empirical evidence of direct costs to sex discrimination—an important avenue for future investigation. Costs to mating preferences from among opposite-sex individuals (Gibson and Bachman 1992; Byers et al. 2005; Vitousek et al. 2007), however, suggest that sex discrimination also may carry direct costs.

Overall, the costs and benefits of expending energy on mating impact the evolution of the mating rate. In classic theory on opposite-sex matings, females may evolve choosiness (resistance) to reduce the mating rate when they suffer from male harm (Holland and Rice 1998; Getty 1999; Gavrilets et al. 2001; Parker 2006). In the context of SSB, high costs to mating favor the evolution of sex discrimination to reduce the mating rate (Table 1). Conversely, low costs to mating reduce the selective benefit to sex discrimination (Allen et al. 2025). An example comes from common toads (*Bufo bufo*), where receiving males emit a release call that leads to the rapid cessation of male-male amplexus and thus low costs to SSB (Marco and Lizana 2002).

On the other hand, opportunity costs to missed matings provide a reason to attempt matings. For instance, the deep-sea squid (*Octopoteuthis deletron*) lives at low density and thus rarely receives opportunities to mate—the resulting opportunity costs to passing up a potential mating may drive SSB (Hoving et al. 2012). This pattern can be predicted from classic theory on mate choice, where high opportunity costs to choosiness (e.g., a risk of going unmated) favor the evolution of less choosy individuals (Fawcett and Johnstone 2003; Dechaume-Moncharmont et al. 2016). To summarize, if missing an opportunity to mate with an opposite-sex individual is costlier than mating with a same-sex individual, then sex discrimination weakens. Analogously, if the cost of passing up an opportunity for a mating is greater than the benefit of nonrandom mate choice, then choosiness for specific individuals of the opposite sex weakens (Table 1).

To my knowledge, only one study with experimental evolution of sex discrimination exists. Sales et al. (2018) reared red flour beetles (*Tribolium castaneum*) for approximately 100 generations at fixed density under strongly male-biased or female-biased sex ratios (9:1). They found that stronger sex discrimination evolved in males reared under male-biased sex ratios. This result matches theoretical predictions that sex discrimination should be most beneficial under male-biased sex ratios, because these are the conditions in which sex discrimination is most likely to alter mating decisions for males (e.g., under female-biased sex ratios, indiscriminate mating by males typically results in opposite-sex matings) (Lerch and Servedio 2021).

Only recently has the origin of sex discrimination been considered (Parker 2014; Monk et al. 2019). To my knowledge, Parker (2014) was the first to explicitly point out that sex discrimination was unlikely ancestral to animals by arguing that a sessile or relatively immobile external fertilizer would not be able to engage in movement-based sex discrimination (i.e., “female targeting”). That said, uncertainty regarding the ancestral condition in animals (Ros-Rocher et al. 2021) and gray areas in what constitutes sex discrimination prevent confident assessment of the origin of sex discrimination. For instance, a process akin to sex discrimination could predate movement if the presence of opposite-sex gametes (or associated chemicals) facilitated gamete release, a pattern with limited empirical support (Zeeck et al. 1998; Fujikura et al. 2007; Caballes and Pratchett 2017). Nevertheless, once movement was possible, selection would act, under many conditions, to favor males that “targeted” sperm release when in the vicinity of females (i.e., to engage in sex discrimination), in a set of evolutionary steps observable in echinoderms (Parker 2014).

The origin of sex discrimination is particularly interesting in light of its coevolution with sexual signaling (i.e., a detectable feature that can be used to identify sex). Theory suggests that indiscriminate mating and the absence of sexual signaling is always a stable equilibrium with any nonzero costs of discrimination and signaling, because there is no benefit to attempting discrimination if the sexes cannot be differentiated and vice versa (Lerch and Servedio 2023; Allen et al. 2025). Thus, if the ancestral state consists of both indiscriminate mating as well as males and females that cannot readily be differentiated, then indiscriminate mating should persist. Given that (imperfect) sex discrimination appears to have evolved repeatedly, it has been argued that detectable differences between the sexes likely arose as a byproduct of the evolution of anisogamy (Lerch and Servedio 2023).

The potential theoretical importance of ancestral conditions could be taken to support arguments that ancestral indiscriminate mating shapes the contemporary distribution of SSB (Monk et al. 2019). However, there are reasons to be skeptical that ancestral indiscriminate mating has widespread implications for SSB today (Clive et al. 2020; Dickins and Rahman 2020). Most notably, there is little connection between the ancestral condition (likely broadcast spawning) and the highly derived courtship behaviors involved in many instances of contemporary SSB (often internal fertilizers). For example, courtship in species like *Drosophila* that are frequently used as a model of SSB (Bailey and Zuk 2009) involve a complex interplay of subtle male and female behaviors that ultimately lead to fertilization (Hall 1994; Lasbleiz et al. 2006). None of these behaviors are ancestral to animals (or even insects). In taxa with highly derived and species-specific mating behaviors, the ancestral mode of reproductive behavior has been lost and thus contemporary SSB is best viewed as derived in these cases (like the mating and courtship behavior of internal fertilizers in general).

## The plastic expression of same-sex sexual behavior

The vast majority of experimental work that has been conducted on SSB considers its plastic expression rather than its evolution. Most of this work involves manipulating the social conditions experienced by insects. I now review how manipulations of both population density and sex ratio have clarified that the expression of SSB is highly plastic. I also explain how direction of these plastic changes can often be predicted from classic theory on mate choice.

Theory on mate choice from among opposite-sex individuals suggests that choosiness is most likely to evolve given frequent encounters (Crowley et al. 1991; Kokko and Mappes 2005; Bleu et al. 2012; Etienne et al. 2014; Henshaw 2018), a result consistent with recent theory demonstrating that SSB should readily evolve at low encounter rates (Allen et al. 2025; Table 1). Empirically, low encounter rates (social isolation) lead to the plastic expression of frequent SSB (reduced choosiness) in many systems (Dukas 2010; Bailey et al. 2013; Engel et al. 2015; Martin et al. 2015). Low encounter rates mean that the opportunity costs of missed mating opportunities are high, resulting in more permissive mating decisions, and thus more SSB (Hoving et al. 2012). Though not universal (Weldon 2005), this result mirrors work from classic theory on mate choice, where more permissive mating decisions (i.e., lower choosiness) evolve at low encounter rates, due to the risk of going unmated (Kokko and Mappes 2005; Henshaw 2018). For example, female viviparous lizards’ (*Zootoca vivipara*) become less choosy after separation from males (Breedveld and Fitze 2015) and female swordtails (*Xiphophorus birchmanni*) become more accepting of a heterospecific mate given infrequent encounters (Willis et al. 2011).

The sex ratio is the component of the social environment that has received the most attention in the context of SSB. Interestingly, the sex ratio does not have a consistent influence on the plastic expression of SSB across species. While the majority of studies find that male SSB is most common under male-biased sex ratios (often controlling for base rates) (Switzer et al. 2004; Svetec and Ferveur 2005; Engel et al. 2015; Han and Brooks 2015b; Macchiano et al. 2018), other studies suggest that female-biased sex ratios promote male SSB (Bailey and French 2012). Others still suggest that being housed with any conspecifics, regardless of sex, prevents the expression of SSB (Dukas 2010; Bailey et al. 2013; Martin et al. 2015). Unlike most work on the plastic expression of SSB, but consistent with theoretical expectations (Lerch and Servedio 2021), experimental evolution finds that males evolve stronger sex discrimination under male-biased sex ratios (Sales et al. 2018).

Recent theory has provided some potential solutions for the apparent differences between how plastic sex discrimination responds to skewed sex ratios compared to how sex discrimination evolves under skewed sex ratios. First, if males are constrained to have few mating attempts in their lifetime (due to, e.g., high mortality or sperm limitation), then males benefit from strong sex discrimination to make the most of their few matings. Consequently, sex discrimination evolves to strengthen upon encounter with either sex, rather than only with one sex, the strategy leading to the strongest sex discrimination (Lerch and Servedio 2025). Second, insects (the taxon typically studied) may learn more strongly from positive, rather than negative, stimuli (Tempel et al. 1983; Unoki et al. 2005; 2006; Honjo and Furukubo-Tokunaga 2009; Nakatani et al. 2009; Schnaitmann 2010). Positive stimuli (like successful copulation; Crickmore and Vosshall 2013; Zhang et al. 2016) are more likely experienced under female-biased sex ratios than male-biased sex ratios where negative stimuli (like aggression; Quinn et al. 1974; Bailey et al. 2013) are more likely. Thus, learned sex discrimination in males may sometimes be strongest under female-biased sex ratios as a physiological constraint.

## Adaptive benefits to same-sex sexual behavior

Above, I reviewed how SSB can be a consequence of imperfect sex discrimination and various reasons that selection may favor imperfect sex discrimination. Other hypotheses for the evolution of SSB consider that SSB per se may be adaptive. Most examples of potentially adaptive SSB involve the co-option of sexual behavior to modify social interactions, either as aggressive or prosocial behavior. These functional explanations for SSB typically provide indirect reproductive benefits and future work more explicitly linking SSB to fitness in these cases is needed. While not always related to classic theory on mate choice, many of the functions of adaptive SSB align with theory on social behavior more broadly.

Before reviewing evidence for adaptive benefits of SSB, I must first clarify what I mean by “adaptive,” since some confusion appears in the literature regarding this point. I refer to SSB as adaptive when the act of expending mating effort on same-sex individuals improves an organism’s ability to survive or reproduce (Box 1). The hypotheses for the evolution of SSB discussed above are not adaptive explanations for SSB per se, because the act of expending mating effort on a same-sex individual is, in itself, not beneficial. Rather, imperfect sex discrimination may be a beneficial mating strategy due to ecological or physiological tradeoffs that exist in a system, with SSB occurring as a byproduct. In other words, adaptive mating strategies may lead to decisions to attempt matings that are, themselves, not adaptive.

Nevertheless, potential cases of truly adaptive SSB exist. The most direct example of adaptive SSB comes from red flour beetles, where males can transfer sperm onto other males, who then transfer the sperm from the first male to females (Levan et al. 2009). While this shows that SSB can be a conduit for indirect sperm translocation, other studies have failed to find evidence of this effect (Martens et al. 2024). Another direct example comes from mate choice copying in Atlantic molly (*Poecilia mexicana*). Small males that are typically unattractive may engage in SSB to manipulate female choice—females are more likely to mate with males that have engaged in either opposite-sex or same-sex matings (Bierbach et al. 2013).

Less direct adaptive benefits of SSB can arise due to its function in intrasexual aggression (Wickler 1967; Dagg 1984). A meta-analysis across mammals suggests that the prevalence of SSB in males is positively associated with the frequency of conspecific killing (Gómez et al. 2023). Dominance-based aggression has been specifically linked to SSB in birds and mammals (Klemm et al. 1983; Jamieson and Craig 1987; Srivastava et al. 1991; Huang et al. 2017). Similarly, in broad-horned flour beetles (*Gnatocerus cornutus*), SSB may serve as a dominance display that prevents costly aggression between males and determines priority access to females (Lane et al. 2016). In other cases, SSB may prevent same-sex rivals from mounting females (Preston‐Mafham 2006) or kill same-sex rivals before they mature (Kureck et al. 2011).

On the flip side, mating can be a tool to strengthen bonds, a pattern known from classic work on opposite-sex pair bonds (Young and Wang 2004; Birnbaum and Finkel 2015; Blumenthal and Young 2023). SSB can serve similar functions in preventing conflict or acting as reconciliation following conflict (Gómez et al. 2023). Most work on SSB in this context comes from primates (Vasey 1995). The bonobo (*Pan paniscus*) is famous for having nonreproductive functions for sex, such as conflict avoidance, tension reduction, and coalition formation (de Waal 1990; 1995; Hohmann and Fruth 2000; Hohmann et al. 2009; Hare et al. 2012; Clay and de Waal 2015). Similar functions for SSB exist in other primates (Fairbanks et al. 1977; Oi 1990; Smuts and Watanabe 1990; Dal Pesco and Fischer 2018; Sandel and Reddy 2021). The potential of SSB to strengthen social ties is not limited to primates, however: SSB among male bottlenose dolphin (*Tursiops aduncus*) coalition partners assists in the formation and functioning of the coalition (Mann 2006) and SSB may reduce tension in crickets (Green et al. 2026). Thus, SSB has repeatedly been co-opted for prosocial functions that appear analogous to nonreproductive functions of sex in opposite-sex pairs (Table 1).

While much discussion of social benefits to SSB comes from mammals, female birds can benefit from cooperating to rear offspring in same-sex pair bonds (an example of SSB distinct from same-sex copulation) (Gillies and Siddiqi-Davies 2025). Classic theory suggests that socially monogamous pair bonds have evolved in birds to provide biparental care to altricial young (Wesolowski 1994; Burley and Johnson 2002; Thomas and Székely 2005; Long et al. 2022). However, in some cases, females may be unable to find a male to contribute paternal care and pair with another female to still reap the benefits of biparental care (Table 1). For example, females in same-sex pairs in a Laysan albatross (*Phoebastria immutabilis*) population with a skewed sex ratio (59% female) have higher reproductive success than solitary individuals (Young et al. 2008; Young and VanderWerf 2014). While same-sex Laysan albatross pairs have lower reproductive success than opposite-sex pairs, classic work on mate choice clarifies that the risk of not reproducing at all (i.e., going unmated) may favor accepting an inferior partner (Fawcett and Johnstone 2003; Dechaume-Moncharmont et al. 2016; Henshaw 2018), analogous to how the risk of a nest failure can favor forming same-sex pairs despite their lower reproductive success than opposite-sex pairs. A meta-analysis across birds suggests that the same benefits of opposite-sex pair bonds (biparental care) lead to same-sex pairs: female SSB is most likely in monogamous species (MacFarlane et al. 2007). In some cases, individuals express a preference for their same-sex partner over opposite-sex alternatives (Adkins-Regan and Krakauer 2000; Elie et al. 2011; Boucherie et al. 2016), likely due to improved coordination when rearing offspring with familiar individuals (Sánchez-Macouzet et al. 2014; Wiley and Ridley 2018; Leach et al. 2020; Wagner et al. 2022). This putative benefit to retaining a same-sex partner also drives mate fidelity in opposite-sex pairs (Lerch et al. 2022).

## Future directions

Clearly, much is already understood about the evolution of SSB, with results often relating heavily to classic work on the evolution of mate choice (Table 1). Still, many important gaps in our understanding of SSB remain. Below, I highlight some of these gaps as well as ways in which classic theory on mate choice motivates strategies for filling them.

Broadly, work on SSB has demonstrated that sexual behaviors can be co-opted for a wide range of functions (Bierbach et al. 2013; Gómez et al. 2023; Gillies and Siddiqi-Davies 2025). Despite being discussed using the same terms, however, it remains unclear to what extent different forms of SSB (e.g., courtship versus pair bonding) actually reflect the same phenomenon. Likewise, it remains unclear what forms of SSB are perceived by the individuals engaging in them in the same way as they perceive the analogous behaviors performed between opposite-sex individuals. While little is known about the mechanisms driving SSB, understanding these mechanisms may shed light on these mysteries. More work is needed to understand the extent to which shared function, mechanism, or constraint leads to different manifestations of SSB. Relevant work could follow Dukas' (2010) finding that simply the presence of other males, rather than being courted by other males, shapes future mating strategies in *Drosophila*, consistent with plasticity being socially cued but not learned from courtship.

Likewise, more work is needed to understand same-sex sexual attraction, which is necessarily distinct from SSB occurring due to imperfect sex discrimination. Theory has differentiated between overdominance (e.g., a heterozygote advantage) or sexually antagonistic selection (e.g., where a gene is beneficial in one sex and detrimental in the other) as two scenarios that can lead to the evolution of same-sex sexual attraction (Getz 1993; MacIntyre and Estep 1993; Gavrilets and Rice 2006). While the determinants of human sexuality are multifaceted and include environmental effects, genes related to same-sex sexual attraction in humans have been linked to overdominance and sexually antagonistic selection (Camperio Ciani et al. 2008; Zietsch et al. 2008; Ciani et al. 2015; 2021; Felesina and Zietsch 2024) (but see Ventresca et al. 2024 for a critique on this body of work). Both forces have long been known to maintain genetic variation in classic theory (Fisher 1930; Rice 1984), demonstrating that the evolutionary drivers of same-sex sexual attraction are neither special nor unique. Many hypotheses for same-sex sexual attraction that do not invoke selection have also been proposed (Blanchard 2001; Rice et al. 2012; 2018; Felesina and Zietsch 2024). Overall, it remains unclear how widespread same-sex sexual attraction is in nonhuman animals and very little is known about how often SSB in nonhuman animals involves same-sex sexual attraction (but see Roselli 2020 for a relevant review in rams, *Ovis aries*).

A greater understanding of sex differences in the expression of SSB is also needed. Most work on SSB (especially in the context of imperfect sex discrimination) considers males. The focus on male SSB likely reflects something real about the underlying biology of the systems studied. Classic theory suggests that anisogamy is linked to the evolution of sex roles: males produce cheaper gametes and thus are less choosy about the expenditure of their gametes (Bateman 1948; Dewsbury 2005; Schärer et al. 2012; Parker 2014; Janicke et al. 2016; Lehtonen et al. 2016; Lehtonen and Parker 2024). Though some have questioned the validity of this argument (Hubbell and Johnson 1987; Snyder and Gowaty 2007; Ah-King 2013), if true, then males are expected to be more likely to court and initiate copulation. Consequently, male SSB may be more common than female SSB, because males are both less discriminate and make the initial decision to attempt mating. While most studies do not assess female SSB, those that do suggest that female SSB is less common than male SSB (Drago et al. 2025) or even a byproduct of male SSB (Burgevin et al. 2013). Still, the lack of consideration of female SSB is an important gap in the literature, and most research on female SSB focuses on vertebrates (Vasey 1995; Gillies and Siddiqi-Davies 2025). Work is needed to understand if male SSB is more common than female SSB and to unify our understanding of sex roles with SSB. Doing so may require using different model systems where it is easier to opportunistically observe female SSB and distinguish it from other behaviors: studying systems in which males actively court females makes it harder to observe female SSB.

Relatedly, it is worth explicitly connecting male SSB to male mate choice, another classic body of sexual selection theory (Fitzpatrick and Servedio 2018) relevant to SSB. Male mate choice is relatively unlikely to evolve in polygynous mating systems (assuming males are not limited by gamete production) because choosy males experience heightened competition for matings and thus produce fewer offspring (Servedio and Lande 2006). This effect can be countered if males prefer individuals that confer higher reproductive success (Servedio and Lande 2006; Nakahashi 2008). Sex discrimination thus may be a form of male mate choice that is likely to evolve because males are choosing between mating with individuals that confer no reproductive success (other males) and those that do confer reproductive success (females).

In my view, the biggest gap in our understanding of SSB is how traits of the individual receiving a same-sex mating attempt influence the expression of SSB. Substantial effort has been devoted to understanding the causes of imperfect sex discrimination and how this leads to SSB. Sex discrimination, however, requires some signal or cue of sexual identity in the opposite sex. Notably, individuals may not be motivated to signal their sexual identity if they are frequently harassed by the opposite sex (Rivera and Sánchez-Guillén 2007; Willink et al. 2019; Falk et al. 2021). A tantalizing example of this comes from *Littorina* snails, where females living in high-density conditions have lost sex-specific cues in their mucus, rendering males unable to preferentially follow female mucus trails to mate (Johannesson et al. 2010). Thus, selection on females to reduce the mating rate may drive male SSB. Future work could also consider whether evolution can drive same-sex traits that trigger avoidance.

While the evolution of signals of sexual identity have recently been considered in theoretical models (Lerch and Servedio 2023; Allen et al. 2025), relatively little is known empirically about what is detected by individuals that are attempting sex discrimination. The few empirical studies that have considered this confirmed that receiver traits influence how likely an individual is to receive a same-sex mating attempt (Han et al. 2016; Richardson and Zuk 2023). Understanding what specific receiver traits affect the expression of SSB is important, however, because within-sex variation in cues used for mate choice could lead to the persistence of SSB even with a strong attempt at sex discrimination. Evidence of mate choice occurring based on variable traits such as pheromone profiles or aspects of body size (Cook and Cook 1975; Scott et al. 1988; Takeda et al. 2020) is therefore important for contextualizing the persistence of SSB.

Classic work on mate choice provides a general framework that can predict how permissive an individual’s mating decisions should be given imperfect signals of sex. Signal detection theory provides a foundation for understanding how individuals can optimally balance the risks of accepting an undesirable mate against the risk of rejecting a desirable mate (Reeve 1989; Getty 1995; Wiley 2006). For example, theory has demonstrated that overlapping mating signals lead to males attempting to mate with unreceptive females (Dukas et al. 2006) and can maintain heterospecific matings (Shizuka and Hudson 2020). This framework is well-suited for explaining how distributions of traits used in mate choice overlapping in males and females could lead to the maintenance of SSB (related to SSB resulting from “broad mating filters”; Richardson and Zuk 2023). Indeed, work on SSB has altered the perceived costs of acceptance or rejection errors and demonstrated the prevalence of SSB changes as predicted by acceptance threshold theory (Engel et al. 2015), providing perhaps the strongest link between classic work on mate choice and SSB in the literature (Table 1).

Finally, while classic work on mate choice is informative for understanding SSB, researchers should consider how work on SSB can inform the study of mate choice more broadly. For instance, SSB is only one example of nonconceptive sexual behavior (Furuichi et al. 2014; Roth et al. 2023) and the causes and taxonomic diversity of such behaviors could be reconsidered in light of studies on SSB. Further, SSB is not the only type of mating that can result from broad mating filters (Richardson and Zuk 2023). The mating-filter approach could be applied to reproductive decisions more broadly and could, for instance, advance our understanding of hybrid zones and reproductive interference.

## Conclusions

Work on the evolution of SSB makes plain that it should not be viewed as an anomalous or unexpected behavior but rather a common component of sexual reproduction. The failure to recognize that opposite-sex mating preferences must evolve and often appear imperfect likely comes from treating them as a “default” rather than recognizing SSB as an expected feature of reproductive decision making ( Monk et al. 2019). That said, there have already been decades of work on the evolution of mating preferences in general and there exist many tight analogs between this classic theory on mate choice and the evolution of SSB (Table 1). Our understanding of SSB, therefore, is greatly enhanced by leaning on this “old theory” and applying it to questions around the presence or absence of opposite-sex mating preferences.

The evolution of SSB provides an excellent case study on the importance of building upon a preexisting foundation to develop deeper insights into a topic. Because this relates to the role of theory broadly (Caswell 1988; Servedio et al. 2014; Smaldino 2017), it is unsurprising that theory has played a key role in clarifying that SSB is no evolutionary conundrum, but rather should be expected (Gavrilets and Rice 2006; Parker 2014; Lerch and Servedio 2021; 2023; 2025; Allen et al. 2025). Indeed, theory demonstrates strong analogs between opposite-sex mating preferences (or the lack thereof) and mating preferences among opposite sex individuals (Fig. 1). In other words, SSB is a twist on many processes that we already understand well (Richardson and Zuk 2023). While there is tremendous value in asking new questions about mate choice in the context of SSB to gain new insights into the nature of sexual behavior, such questions have strong precedents and a firm theoretical foundation in the mate choice literature.

This is notable because SSB is sometimes presented as an almost shocking phenomenon that makes no sense given our understanding of evolutionary principles. However, as we have seen in the above review, that view is incorrect: we have a very good understanding of many of the ultimate drivers of SSB. Indeed, not only do we have a good understanding of why SSB evolves, but that understanding also has much deeper roots in classic work on mate choice. There exists a perception that peer review is a conservative process that prevents novel ideas from entering the scientific literature. Evidence suggests otherwise. Manuscripts are more successful in peer review when they present results that are more novel (Teplitskiy et al. 2022), which may create pressure to overstate novelty and interfere with understanding the natural world (Smaldino and McElreath 2016). Our understanding of SSB in particular and nature in general depends on our ability to disconnect from our favored hypotheses, contextualize our results appropriately, and rigorously and critically evaluate each decision that leads to our results.

*“The first principle is that you must not fool yourself and you are the easiest person to fool.”*

- *Richard Feynman*

## Supplementary Material

icag015_Supplemental_File

## Data Availability

This manuscript does not use data.

## Notes

From the symposium “Sex across origins: Questioning animal-centric assumptions and developing integrative frameworks organized by A. M. Aramati Casper, Colorado Sate University Jay J. Falk, Smithsonian Tropical Research Institute Karen M. Warkentin, Boston University and Smithsonian Tropical Research Institute” presented at the annual meeting of the Society for Integrative and Comparative Biology, January 3-7th , 2026.
